# Spirituality and Children’s Coping with Representation of Death During the COVID-19 Pandemic: Qualitative Research with Parents

**DOI:** 10.1007/s11089-021-00995-w

**Published:** 2022-02-11

**Authors:** Sara Pompele, Valentina Ghetta, Serena Veronese, Mihaela Dana Bucuță, Ines Testoni

**Affiliations:** 1 Department of Philosophy, Sociology, Education and Applied Psychology (FISPPA), University of Padova, Padova, Italy; 2 Lucian Blaga University of Sibiu, Sibiu, Romania; 3 Emili Sagol Creative Arts Therapies Research Center, Faculty of Social Welfare and Health Sciences, University of Haifa, Haifa, Israel

**Keywords:** COVID-19, Children’s representation of death, Religiosity, Mortality salience

## Abstract

The coronavirus disease 2019 (COVID-19) pandemic and the related lockdown measures have had intense negative impacts on the psychological well-being of both adults and children. Among such impacts is a significant increase in mortality salience and changes in how people deal with grief and losses. This qualitative research used semi-structured interviews with 23 Italian parents to draw insights on the impact of the pandemic on children aged 5˗15 years with regard to their representation of death and the eventual role that family spirituality/ religiosity played in helping them understand both the concept of dying and possibly the pandemic itself. From the data analysis, four main thematic areas emerged: “Lockdown experience,” “Fears and worries related to COVID-19,” “Emergence of thoughts on the process of dying,” and “Representation of death and the impact of religious beliefs.” The participants highlighted how stressful the lockdown measures have been for their children and the anxiety that their children have experienced because of fears related to the pandemic. The interviews also surfaced how living in a religious family has contributed significantly to shaping children’s representation and understanding of death and sometimes even helped both the parents and their children to face difficult moments such as those caused by the pandemic.

The current coronavirus disease 2019 (COVID-19) pandemic has had intense negative impacts on children’s psychological well-being. For example, rigorous lockdowns to contain the pandemic, such as that in Italy from March 9 to May 18, 2020, that completely blocked people from leaving their homes, have disrupted children’s daily habits and deprived them of physical contact with classmates, friends, and teachers; of personal space at home; and of opportunities to pursue their interests and hobbies, which have led to their frustration and boredom (Demaria & Vicari, 2021; Wang et al., 2020). Indeed, many studies, some of them conducted during this pandemic (Orgilés et al., 2020; Spinelli et al., 2020) and others during similar situations of high public health risk (Sprang & Silman, 2013), have highlighted how containment measures such as isolation can be traumatizing for both children and their parents.

Other possible significant impacts of the pandemic on children’s psychological well-being are linked to the inevitable increase in mortality salience, that is, in elements that every day remind of the theme of death and human vulnerability, consequent to the constant news of seriously ill and dying people because of COVID-19 (Pyszczynski et al., 2021). This, in turn, has led to an increased perception of personal and loved ones’ risk to suffer serious health consequences, in parallel with a highly increased chance of directly experiencing bereavement for the first time by more children (Albuquerque & Santos, 2021).

In addition, due to COVID-19, there have been enormous changes in the way people deal with death and grief (Diolaiuti et al., 2021). In many Countries, including Italy, people have for example been forbidden to visit relatives in hospitals and even to properly celebrate funeral rites (Wallace et al., 2020).

These changes have led to an increased distance between people and the concrete aspects of the end-of-life, for adults and even more for children who, even before COVID-19, had already been frequently excluded from such ceremonies in a society that finds it extremely difficult and puzzling to deal with the theme of human vulnerability and death (Testoni et al., 2021).

The idea of death itself is not a simple notion, but instead, a complex system of sociocultural beliefs, personal experiences, and religious cues (Slaughter, 2005), and thoughts and ideas concerning it start to develop rather casually during childhood, based on the kind of information the environment offers (Longbottom & Slaughter, 2018). Therefore, opportunities to explore the theme of death can have an important influence on children’s way of perceiving it and even on their ability to deal with future eventual losses (Renaud et al., 2013).

However, despite the importance of allowing children to explore the idea of death, parents often have difficulty talking about it with their children (McGovern & Barry, 2000; Testoni, 2015). Many parents consider themselves incompetent to handle such discussion (Slaughter, 2005) and fear that they would somehow cause their children anxiety (Melvin & Lukeman, 2000).

Another basic factor of people’s handling of the theme of death, which also determines the way adults talk about it to children, is their ontological representation of it–-that is, either as a passage towards a new dimension or as total annihilation (Harris, 2018; Testoni et al., 2016). Studies have indeed shown how being religious and believing in life after death, or even simply having a personal form of spirituality, can help people face conditions of extreme vulnerability, such as serious illnesses or organ transplants (Musarezaie et al., 2014; Testoni et al., 2017) and even the end-of-life (Candy et al., 2012; Testoni et al., 2018). This element has also emerged for children, who have spiritual needs as well and tend to adopt religious coping mechanisms especially when facing grief, serious illness, or the end-of-life (Hufton, 2006; Pendleton et al., 2002). In this regard then, having a concept of death as a moment of passage towards another existence, in comparison to considering it the end of everything, seems to contribute to protect people more, even children and adolescents, from anxieties and anguish linked to the idea of dying, as has been highlighted by some experiences of Death Education (that is, programs of education that promote reflection and dialogue concerning the very theme of death and dying, the related emotions and fears, and the concept of spirituality and afterlife) implemented with children of different age groups, from kindergarten to high school (Testoni et al., 2019a, 2020b).

Since a very important source from which children elaborate their perception and representation of death and of an eventual afterlife are usually their parents, it is possible, as it has already been observed and reported in literature (Panagiotaki et al., 2018), that parents who present a religious view or even simply a personal form of spirituality that leads them not to consider death as total annihilation could inspire such a view in their children as well, consequently allowing them to better manage their eventual death anxiety or the loss of a loved one (Zajac & Boyatzis, 2021).

It is thus understandable why, in historical periods such as the present one, characterized, as noted before, by an unexpected pandemic that has made the concept of human frailty and death much more salient, these personal and sociocultural factors need to be considered and could have an even stronger impact on children’s representation of death and their attitude towards it. Moreover, since literature has shown, as it has been previously mentioned, that spiritual or religious kinds of coping can be adopted by children as well with beneficial effects, even in conditions of serious illness, (either personal or of a loved one) (Cotton et al., 2012; Lövgren et al., 2019; Reynolds et al., 2016) it is possible that such coping mechanisms could prove to be useful even in a situation such as the present one of global pandemic, another important aspect that should be taken into consideration.

Against this theoretical framework, the present research has been conducted in order to explore these important matters and how they eventually related to one another. More specifically, the authors aimed to investigate the following themes:

1. The way children faced the lockdown period and whether the theme of COVID-19 affected their emotions and how;
2. Whether the children had already thought about death before the pandemic, and, if they did, what their representation of it was. In this case, the researchers also explored whether such a representation had changed because of their experience of the pandemic, how the parents had felt having to explore such a theme with them, and how they had helped their children deal with it;
3. Whether the participants (the children’s parents) believed in an afterlife, or had a personal religiosity/spirituality, and whether and how it had helped them support their children in adapting to death in general and, eventually, in facing the unexpected lockdown period.

## Materials and Methods

### Participants

Twenty-three Italian parents (20 female and three male) of children aged 5˗15 years participated in this study. Their ages ranged from 36 to 47 years (mean = 42, SD = 4.2).

One participant had four children; seven participants had three; 13 participants had two children; and two participants had only one child.

Sixteen participants had a high school diploma, and the rest had a university degree.

The authors chose to recruit parents instead of directly interviewing children for multiple reasons: firstly for practical ones, since the interviews, because of the health risks linked to COVID-19, had to be conducted online (through platforms such as Zoom or Skype) or by phone call, and given the high variability in the children’s age (with the youngest ones being not older than five years old) the necessity to sustain a 40 min long interview on complex themes at distance (a mean that could hinder children’s ability to concentrate) could have represented an obstacle for them, therefore, parents were chosen instead.

Moreover, another reason was instead more related to the very nature of the research questions, and more specifically to the ones that aimed to investigate whether the children had thought about the theme of death before the pandemic, as well as to the ones that explored parents’ beliefs in the afterlife and their response (not only in terms of what they said to their children but also concerning their own emotions and thoughts) to children’s questions concerning dying. It was indeed considered potentially too complicated for children, especially for the younger ones, to be able to adequately grasp and focus on their parents’ emotions and thoughts concerning these themes (which could even have been kept hidden to them by their parents). Moreover, more importantly, very young children might not have been able to fully remember whether they had already encountered the concept of death in their life and asked about it to their parents, which was indeed a very important aspect to explore for the present research.

Lastly, the idea to ask young children to reflect upon their parents’ representation of death and of the afterlife seemed too complex as well, since parents could have not spoken to them openly about it yet. Therefore, since the authors wanted to possibly explore not only older children’s points of view but younger ones’ as well, they deemed it more appropriate to interview their parents instead, in order to be able to receive exhaustive explanations concerning all the specific themes that needed to be explored for the study.

The participants were either people whom the researchers knew personally or referred by the initial recruits. The researchers thoroughly briefed all of them online on the research objectives and methodology.

This research followed the American Psychological Association’s Ethical Principles for Psychologists and Code of Conduct, and the principles of the Declaration of Helsinki. It was approved by the Ethical Committee for Psychological Research of the University of Padua (Italy) (n. FF689133424358CFB47209297ADD91AA). Each participant’s signed informed consent was obtained.

### Data Collection and Analysis

A qualitative methodology was used (Denzin & Lincoln, 2011), since it was deemed the most appropriate one to allow an exploration of single participants’ points of view as in depth as possible, presenting to the readers their direct words, thus allowing people to fully grasp what these experiences meant to them, a result that cannot be achieved through quantitative research, which is instead more indicated when the purpose of the study is to produce generalizable statistics over a phenomenon. Instead, the researchers aimed not to test a pre-determined hypothesis but elaborate concepts and theories from empirical data on an observed phenomenon, following the principles of reflexive Thematic Analysis (Braun & Clarke, 2006; Testoni et al., 2019b), a methodology that, through an inductive, bottom-up approach, allows to identify new categories and patterns of meaning that are peculiar to the research participants. Therefore, one of the peculiarities and benefits of this perspective was the active position of the researchers while they interacted with data, which allowed them to understand the genesis of analytical categories and the identification of relations between such categories. During the process, researchers maintained a flexible approach, remaining faithful to the information provided by the collected data, without imposing their pre-constituted cognitions or points of view.

Data were collected through 40 min-long semi-structured interviews or conducted on the phone or through online meeting platforms such as Skype, Zoom, etc. Each interview explored the lockdown experience, children’s emotions related to it, the questions they asked their parents, eventual themes linked to the concept of death in their everyday talk, whether the theme of dying had already been discussed in the family prior to COVID-19 or not, and how the concept of death was presented to the children. Since they were semi-structured, the interviews did not follow a strict template, but rather they were flexible and adapted to each participant’s interests and narrated experiences, so that the person was able to explore more in depth the concepts and ideas that he/she felt were most important, though the interviewer was still careful to investigate all the reported themes.

Each interview was conducted in Italian, the participants’ and authors’ native language, and recorded (after the participants specifically and explicitly agreed to the recording). Then the recording was transcribed verbatim in a computerized text file. The transcriptions were analysed following with six main phases: engaging in preparatory organization, reading the texts deeply, coding data, interpreting themes, searching for alternative explanations, and producing the final report (Testoni et al., 2020a). In this process, the researchers start from a single interview and identify salient concepts (expressed by recurrent words, expressions, ideas, emotions reported by participants), coding them with specific labels. Subsequently, all the conceptual labels assigned to each interview text are compared, in order to identify common aspects and general themes, while the eventual relations between them are analysed and a proper identifying code is assigned to them as well. In the final steps of the process, the researchers review the analysed texts and the main themes emerged and elaborate broader thematic categories that can encompass all the fundamental meanings that are commonly shared between the different texts (Testoni et al., 2019c). The analysis was performed with the support of the software *Atlas.ti* (Muhr, 1991), which has been specifically designed to aid researchers in thematic analysis of written texts.

After the analysis, the participants quotations have been translated in English, for the purpose of writing the present article. The authors, who all possess at least a C1 degree in English, have been extremely careful in their translation, in order to maintain unaltered the original meaning of each quotation, and they have been closely working with a bilingual translator who knew the aim and themes of the research and discussed with them the precise meaning of each sentence and the best possible translation in English. Lastly, as a final check, the authors have also asked some of the participants (those who had at least a B2 degree in English) to check the translation of their interview and to confirm whether they believed their original intended meaning had been maintained.

## Results

From the data analysis, four main thematic categories emerged: “Lockdown experience,” “Fears and worries related to COVID-19,” “Emergence of thoughts on the process of dying,” and “The representation of death and the impact of religious beliefs.”

### Lockdown Experience

Concerning the children’s experience of the lockdown period, the parents relayed a rather complex and contradictory situation, with an initial prevalent happiness because of the chance to avoid school and spend more time at home, and the later increasingly difficulty of accepting the strict rules imposed on them.

Ludovica, a 46-year-old married woman with three children aged 15, 13, and 8 years, shared how happy her children were at first, especially the youngest:

[She] took it well; in fact, she could enter another lockdown period right now, because she spent all day close to me. She had her mom just for her, so you can imagine... she took it very well; she was extremely happy.

Giulia, a 41-year-old married woman with two children aged 10 and 12 years, said her children considered the lockdown wonderful news at first, but after their initial enthusiasm, they started to become very weary of the situation:

At first, there was so much happiness because they were at home, they did not have to go to school, they had taken it as a vacation. After some time, however, when they realized they were not going to return to school soon and that they could not even maintain all the sports and other activities they used to conduct on their free time, they started to ask when they could return to school, they started to pose some questions concerning the situation, they started... not really to worry but to say they would have preferred to return to the previous situation.

Many parents indeed expressed how, after a while, their children became tired of the strict lockdown limitations. Their lessons at a distance through the computer posed another intense challenge.

Lisa, a 36-year-old married woman with three children aged 7, 4, and 15 years, highlighted how difficult distance learning was especially for her oldest son, who felt very isolated:

He stayed in his room. Not exactly all day but almost all day, he had to stay in his room because he had distance learning activities, and then in the afternoon, he had to do his homework. You know, he is attending an art school, so he also had a lot of drawings to create. So he practically spent all the lockdown period confined in his room, then once in a while, when he couldn’t take it any longer, he went outside in the garden to read a book for a while. He spent the lockdown like this.

Maria, a 47-year-old married woman with two children aged 10 and 12 years, added:

For them, the biggest trauma has been distance learning, having school but at home. It was a stressful situation for everyone, us parents included because we had to deal with a role that does not pertain to us, because... in a context that is different from the one of school, they felt free, they thought they had all the time they needed to do their homework, but instead, there were deadlines.... And they had a sort of reject for it. Therefore, it has been difficult to manage their attitude towards distance learning.

Another intense difficulty that many participants pointed out was the abrupt interruption of their children’s social life and of their opportunities to maintain their relationships with their peers and friends, which destabilized them significantly.

Mattia, a 37-year-old married man with two daughters aged six and three years, expressed how difficult it was, especially for his first daughter who had already experimented on the typical social life linked with school attendance, to accept the lockdown restrictions:

Well, I’d say it was very different for my older daughter than it was for my youngest. The oldest, who already went to school before the lockdown and who is also very outgoing and likes spending time with other kids, she has suffered a bit, especially [due to] not being able to go to kindergarten, to play with other kids. She missed them, so she asked us, ‘Why can’t I go to school?’ So we tried to explain it to her; we told her there is this virus, which is not dangerous for little kids like her, mostly for the elders, so we have to stay at home, we cannot go outside and maybe spread it.

Alessia, a 37-year-old married woman with two children aged one and five years, also shared how her oldest son suffered intensely because of the loss of his social role in school and of all his relationships with other important figures such as his teachers and classmates:

He suffered a lot; he did not show it much, but he suffered, especially the lack of his social role, which is being at school, with his teacher, with his classmates, interacting with his peers; and he missed that terribly, he still misses it. It was an inner pain; he did not have any hysterical or rage crises, it was an inner suffering because of lack of interaction, so he manifested it through fear of sleeping alone, being alone on the first floor of our house, playing alone˗˗all was related to his fear of having lost his social role.

### Fears and Worries Related to COVID-19

Besides the impact of the lockdown measures, including the abrupt change in their children’s daily habits, the participants expressed how the idea of COVID-19 and of the danger it represented made its way into their children’s thoughts and caused many of them to experience increased anxiety.

Margherita, a 41-year-old married woman with nine-year-old twins, explained this very clearly:

My children do not really pose questions about the situation, but rather, they are scared.... Last week, for example, they waited for me to get home after I had a test for COVID-19 done and they asked, ‘So, are you negative?’ and that surprised me; I mean I have been negative for a year, now I am also vaccinated, but they asked it. [At] other times, they ask me: ‘Is wearing a face mask enough? Will this end? When?’.

Bianca, a 37-year-old married woman with two children aged 11 and 8 years, highlighted her children’s worries as well:

Well, yes, yes, they were worried especially during the first months of the pandemic, because they had no idea what could have happened. At the time, all you heard was people being sick and who had to go to the hospital.... During the first cases, my children seemed very scared.

Lucia, a 36-year-old married woman with two daughters aged 10 and 12 years, added that her youngest daughter was also particularly scared of the effect COVID-19 could have on her grandfather, to whom she is very close, so she demanded that he always wear a face mask to protect himself:

She was also worried for her grandparents. Especially her grandfather, who did not use a face mask often, and we found it difficult to make him understand the importance of it. So, she went to him and said: ‘Won’t you put on your face mask? Do you understand that you have to wear it? Because if you don’t, you die; can’t you understand it? You will die if you don’t!’ or even: ‘Did you wear a face mask when you went outside? Did you have it? You were talking with that man, and you had it here, under your nose. You need to cover your nose as well!’.

Concerning what could have contributed to the increase in their children’s worries and fears related to COVID-19, many parents stressed how media, especially television and its daily news on the pandemic, probably had a considerable role in it. Lucia said:

We kept the television news turned on all day at first, and I started to see my youngest daughter getting bogged down. I mean that she stayed on the sofa and did not want to play; in fact, if someone changed [the] channel, she protested because she said we needed to be super informed on the virus.... She was very scared because there were all those dead people, she did not want to play or to see anyone, because she had to constantly monitor the situation on the news.

Margherita expressed the same concept, highlighting how bothered her children were because of the constant catastrophic COVID-19 news on television:

Well, let’s say they felt a lot of pressure because of these special editions of the news on television; they were very unsettled. It often happened [when] I was lying in bed after dinner, especially with Francesco [one of the twins], and these special editions of the news would start, and he told me: ‘Change channel because I do not want to hear about this anymore.’

Alessia also explained how impactful hearing news on COVID-19 was for her older son:

We do not usually watch television by choice. He only watches some cartoons once a week; he is completely detached from the reality that mass media describe.... However, he is a child who listens a lot to what is happening around him; so if, for example, we are in the car and there is the news on the radio, he interiorizes a lot what he hears. And indeed, because of this, he is terrorized; he developed these fears of being too close to his schoolmates, [of] interact[ing] too much with them, [of] be[ing] close to people he does not know.... He has interiorized this insecurity towards other people.

### Emergence of Thoughts on the Process of Dying

Concerning the theme of death, all the parents said their children had already come in contact with it prior to the pandemic, although almost exclusively in a vague way, as, for example, because of the death of a distant relative or simply because they heard their parents talk about it. Fortunately, none of them had suffered the loss of a loved one because of COVID-19. Because of this, though, some participants, especially the ones with younger children, said their children probably did not have a clear idea of death yet.

Stefania, a 47-year-old married woman with two children aged 12 and 9 years, stated:

Well, yes, they have come in contact with the idea of death, most of all because my mother is very old. She is old, alone, so we often talk about it: ‘When grandmother will die....’ However, frankly speaking, my children are too young; they still do not realize what it implies. They haven’t been to funerals yet and... they do not understand what the real meaning is, that is, when you lose someone, when he/she is not there anymore.

Gaia, a 38-year-old married woman with five-year-old twins and an eight-year-old daughter, expressed the same concept:

My children, they never faced this theme of death, I mean, not because they asked about it. Sometimes, we talk about it; for example, we say, ‘Grandfather died....’ However, they do not... they still do not have this concept in their head.

However, many participants, even though their children have had no direct contact with death yet, shared how they still believe such a theme is crucial because it is part of life itself, and that they have talked about it with their children on some occasions. Denise, a 33-year-old married woman with two children aged three and seven years, said:

When their two great grandmothers died... my son, who was older, came to the funeral; he saw his great grandmother dead, he touched her and all. So we have always let them free concerning the theme of death, I mean, I’ve never hidden this aspect from them because I believe death is part of life, it is the other side of the coin, so it is only right [that] they know it, they see it, especially because years ago... death was much more.... Since people often died at home, it was part of life, while now, since we all end up at a hospital, it is as if death is another dimension, don’t you think? While on the contrary, I believe it is right to know it, since sooner or later, it happens, it is right not to be completely unprepared, but instead to have some awareness of it since we are young; we should not hide it.

Giulia agreed:

We have always treated this theme as any other topic. I mean we did not go in search of talking about it but yes, it happened that the grandfather of a schoolmate died, or there has been, for example, an accident during which the mother of a kid at school died, so we talked about it. They do not bring the theme to the discussion themselves normally; however, if it happens, we talk about it.

Nonetheless, some parents revealed that for their children, the idea of dying could be scary. Giovanna, a 34-year-old married woman with two daughters aged one and five years, said her oldest daughter found the representation of death on screen, through cartoons such as, for example, The Lion King, almost unbearable, while, on the other hand, she easily tolerated the idea of the loss of a relative prior to her birth:

We started to watch The Lion King once, and she said: ‘Mom, why is it like this?... It’s ugly; that is not right.... No, Mom, turn it off, please. I don’t like it.’ You could see tears in her eyes... it must be because she is not ready yet; I don’t know. It is something stronger than her.... We talk about death sometimes; for example, we talk about my mother who died before she was born, but we always think about her, you know. Sometimes, we go to the cemetery, bring her a flower, or we drive close to it in our car, and she says: ‘Hello, grandmother.’ We always talk about it in a light way, so to speak.

Lucia’s case was more drastic. She expressed an intense reluctance to talk about death with her older (12-year-old) daughter. Her reluctance was not a generalized one, though, but was due to her fear that her adolescent daughter would not fully understand her and could thus be at risk of suicide:

I do not really feel like discussing the theme of death right now, for the simple reason that we also have an adolescent teen, and adolescence is now not at all the one we parents experienced... so talking about this today with everything you hear about the adolescence phase, I don’t feel like it.... Nowadays, self-harm is fashionable, and they haven’t understood anything; ignoring it is useless... we are failing as parents because we feel we must conform to society, to a society that has nothing to give to our children.... So, if I discuss death now [with my adolescent child], with everything [that] society presents our children, with the idea [that] adolescents should challenge it, when I face this theme of death now, with an adolescent girl who has a difficult road in front of her, I fear I might do harm.

Lucia thus offered a peculiar point of view that no other participant had presented–-the intense fear that talking about death could harm one’s children, who are probably not ready to think about it properly.

However, when questions about dying did emerge, some participants tended to offer rather concrete explanations, even though they always tried not to scare their children with too many unsettling details, as Alessia described:

Yes, the theme of death has emerged with my son, mostly because he is the kind of child who asks a lot of questions, so he asks me: ‘Are your grandparents dead? How did they die? And where did they go?’... When he asks this, I tend to tell him the truth. I realize that perhaps he is too young... so I can avoid certain things, for example, the fact that a dead person’s body is devoured by worms.... I personally tend to tell him exactly how things are, so I say that when we die, we stop breathing, our body stops living, those who believe in Jesus believe there is a soul that goes somewhere, however, we always carry our deceased loved ones in our hearts; that’s it. I tend to give him this description that is rather realistic but not too much because, I mean, he is still young anyway.

Alessia was also one of the few participants who noted that her child’s questions concerning death have somehow also been influenced by the pandemic:

Yes, he does ask more specific questions during this pandemic period, for example, ‘If this virus only provokes a cold, why are people dying because of it and we have to stay at home?’ So, we had to explain to him a little how illnesses work... and another thing that has emerged is linked to the fact that he has two names, one of which he was given because an aunt of his died while we were waiting for him to be born so he asked me, ‘So my aunt had coronavirus?’ So, he went to look in his memories to understand what kind of relation this situation could have with an experience he had lived.... So now, he has all these questions going on in his head and he lets them out when you least expect it, but we try to satisfy his requests.

### The Representation of Death and the Impact of Religious Beliefs

Finally, another topic closely related to death that emerged from the participants’ interviews was the children’s representation of death and of the afterlife. While describing their children’s first contacts with the idea of dying, many participants also narrated how they usually tried to answer questions related to the theme. Almost all of them revealed that they offer a religious explanation, because of which their children usually have a religious (Catholic) interpretation of death and life after death, although a very simple one. Many participants said such religious explanation is their choice because they are believers themselves. Bianca expressed:

We absolutely give a religious perspective to our children, and we have indeed talked about the theme [of] heaven-purgatory-hell recently. Death is linked to Christianity in our family. Our children know [that] people who do not behave well go to hell; we have talked about this.

The same has been reported by Gaia**,** who also described how she sometimes reads and explains the meaning of some prayers to her oldest daughter:

Just a few hours ago, we were saying our prayers and... I actually explained to her the meaning of eternal rest. I was really explaining to her the meaning of ‘Eternal rest, grant them, Lord, and let your perpetual light shine on them,’ and she asked, ‘But what does *the perpetual light* mean?’ So I explained it to her a little and she said, ‘It’s so beautiful, mom, I can’t wait to be there.’ So yeah, just a few hours ago, with this prayer, we came to discuss the theme of death a little and she... she imagines it like this; at the moment, she has this idea that is rather Christian.

Maria has confirmed too that religion is an important part of their life and it is the means by which she can deal with the theme of death with her children:

I use a religious view to talk about it. They know, they know we are born, and we die. Obviously, religion and faith are present, and they help us a lot, not just them who are children, but us adults as well, you know.

For Maria, therefore, religion represents also a concrete and significant help in moments of difficulty.

Mattia confirmed his belief in the fundamental importance of having a personal spirituality and, in his case, a religious fate, while dealing with children’s questions concerning death, since he would feel somehow lost without a religious perspective on dying:

My daughters surely ask me questions concerning death, as all children do, and we surely must give them an answer somehow; and so, religion, spirituality, and faith are important instruments we have to answer them and I would not know how to do it without them.... For example, we often go to the cemetery to meet my grandparents, and today, my daughter Anna saw [that] they were digging up some old tombs in the first part of it, and she asked me, ‘Where do they go now, those people who were there?’ So I tried to answer her that once you are dead, your body is not important anymore, the body is nothing anymore, it disintegrates in the earth... and that what goes to heaven is not the body but the soul.

Mattia also highlighted how he believes giving children such a perspective and interpretation of dying could be fundamental in allowing them to become strong enough to properly reflect on such matters without too much anxiety and to deal with future painful moments in life:

I believe it is important from a human point of view to give them a reassuring message, because if you don’t, they could think about it in a way that could scare them even more than the answer you give them and, on the other hand, I think that if you manage to give them a message full of faith, if you do it when they are still young without forcing it, just in the occasions during which you have the chance to reflect on this with them, that could be one of those things you can continue to possess in your life even when you are older.... For example, when there is a difficulty, a challenge, or an illness, instead of becoming desperate as if there is nothing else, you are able to face it in a different way. And so when I tell them that ‘not everything on this Earth, not everything ends with this life, there is not only what we can see,’ it is an important lesson for them because I believe that when they will be older and will have to face such things, they will remember this.

Lucia had the same opinion. She not only agreed that a religious view is how she explains death to her children, but she also found support in religious practices she invented at home and involved her children in during the lockdown period:

We use a religious interpretation often because we are Catholics, devout, so... it is something that is part of our everyday life.... The fact that there is a heaven, with angels, that after death, people, for example their grandparents who are not here anymore, they go to a beautiful place.... During the lockdown, we restarted our old habit of sitting all together at 8 p.m. and saying our prayers together, my husband and our children too. We obviously believe praying is fundamental, and so is telling our children that science can arrive up to a certain point but if you ask for God’s help, you arrive at your destination way faster.... We knew how many people were dying, so in the evening, during our prayers, we created some post-it notes; everyone would write on a post-it note a prayer and we read it to each other, and we then hung it on God’s cross. Our objective was, once the pandemic ends, to tie them to seven balloons with the colours of the rainbow and let them fly. Unfortunately, we still have them in our garage since the pandemic has not ended yet.

## Discussion

This study surfaced the negative impacts of the COVID-19 pandemic and of its related restrictive containment measures on children aged 5˗15 years. The main difficulties that emerged were linked to the children’s forced social isolation and lack of contact with their peers and friends as well as their inability to pursue all their typical leisure activities before the pandemic. Another intense challenge that Giulia, Lisa, Maria, Mattia, and Alessia pointed out pertained to distance learning. Since schools had been closed for months, the children had to adapt to online lessons and to the indirect presence of their teacher.

This challenge also appeared in recent literature on COVID-19 consequences on children’ psychological well-being (O’Sullivan et al., 2020) and other studies conducted over the years in similar lockdown conditions due to high risks to public health (Sprang & Silman, 2013).

Another aspect that emerged was the emotional toll on children of the unusual concept of a dangerous virus. Margherita, Lucia, and Bianca said their children feared for their health or for their family members’ health, feared losing their loved ones, and felt a common sense of uncertainty and anxiety.

In such a complex context, some participants (for example, Lucia and Alessia) said the media, with their daily reports of infected and dead people, have contributed to the increase in their children’s uneasiness and anxiety. In one case, watching such news led to behavior similar to depressive symptoms (Lucia’s daughter refused to play and continued to watch the news in a state of sadness and anxiety). The intense impact of media on children’s attitudes toward COVID-19 has already been highlighted in literature as well (Drouin et al., 2020).

In a historical period in which mortality salience has increased significantly, this research also investigated whether the theme of death had already emerged during pre-pandemic conversations of the participants with their children, or if it had been stimulated for the first time by a reflection on COVID-19.

The study revealed that almost all the participants had already had the opportunity to discuss the topic of death with their children, even though none of the participants’ children had experienced losing people very close to them. Indeed, the topic of death surfaced with their children in a casual way, stimulated by indirect experiences. This finding has also appeared in current scientific literature, since children’s idea of ​​death generally starts to develop from their casual experiences (Longbottom & Slaughter, 2018).

However, some participants (for example, Alessia) noticed how their children have displayed greater interest in the subject of death during the lockdown period by asking more specific questions concerning the concrete risks of death because of the virus.

The participants who had little children (for example, Stefania and Gaia) stressed, though, that their children cannot yet fully understand all the implications of the end-of-life. This is also supported by literature, which discusses how the concept of death develops and becomes more and more mature as children grow (Panagiotaki et al., 2018; Slaughter & Griffiths, 2007).

Another significant finding of this study is that when children ask questions concerning dying, only one participant, Alessia answered with a more concrete perspective, describing the biological process of the cessation of life, while others (such as Lucia, Bianca, Gaia, Maria, and Mattia) tended to resort to a religious perspective, specifically Christian-Catholic, due to their intense personal faith.

Given this aspect, it is understandable how the representation of death prevalent among the participants’ children was linked to a religious perspective. Therefore, death was explained as a passage towards a better life.

Almost all the participants believed it was appropriate and important to freely talk about death with their children since they considered death a natural part of life (for example, as Denise and Giulia expressed). This belief could be partly linked to a religious vision, which makes death appear less terrifying because it is not seen as leading to total annihilation (Solomon et al., 2017; Testoni et al., 2016). Only one participant, Lucia, expressed fear of talking explicitly about dying with her adolescent daughter, whom she feared could then be more inclined to commit suicide.

The participants’ general openness to the topic of death was also tied to the fact that most of their children did not perceive death as particularly distressing, partly due to their young age and thus, their lack of complete understanding of death, but also due to their parents’ serene representation of the afterlife, as indicated, for example, by Gaia.

This has been reported by other studies as well (Hufton, 2006; Zajac & Boyatzis, 2021). The studies found that the more people talk with their children about death without inhibitions, the more serenely their children can deal with death. The studies further showed that children who are given an ontological representation of death as a passage towards something better rather than as total annihilation are generally more serene in the face of death.

For one participant (Lucia), being religious also gave her a tremendous source of support during the lockdown period, since it allowed her to be even closer to her family and to take on activities that could help her children cope with the need to remain at home.

Overall, the participants expressed well the need to maintain an open dialogue with children concerning the end-of-life. Parents can be supported in providing their children proper death education by helping them to discover their own spirituality, so that they can find more meaning in both life and death (Mcgovern & Barry, 2000; Testoni et al., 2020c).

### Conclusions and Limitations of the Study

In this study, parents revealed that their children have lived the lockdown period both with initial joy and subsequent difficulties (especially related to distance learning and the impossibility to spend time with friends and peers), and that the COVID-19 had a significant impact on their children’s psychological wellbeing, with many of them expressing fear and worries related to their loved ones’ health. Parents have also reported that their children had indeed developed a personal idea of death and of the afterlife before the pandemic, and it was generally a rather peaceful, non-terrorizing one, mostly linked to a religious (Catholic in particular) representation of existence, since that is the way most parents had tried to explain death to them, maintaining an open dialogue concerning it. Their representation of death did not change particularly because of COVID-19, even though some children did express more questions and doubts specifically related to dying because of the COVID-19 virus. Lastly, most participating parents expressed their own spirituality and religiosity and some of them also highlighted how this had helped both themselves and their children face the lockdown period and the fear related to COVID-19 (as well as other moments of grief).

This study suggests therefore the need to maintain an open dialogue with children concerning death. It also encourages parents to let their children explore their personal spirituality and eventual religiosity in order to help them form a positive, non-terrorizing representation of death especially during historical periods characterized by high mortality salience, such as the present.

This study had some limitations. It had a small sample size, a typical characteristic of qualitative research that hinders generalization of the results. Moreover, only parents were interviewed on the psychological impacts of the pandemic on their children, instead of the children directly. Finally, the children who were the subjects of the interviews had not yet directly experienced the loss of a loved one or deaths due to COVID-19. Future studies could broaden the number of participants possibly include interviews directly with children and investigate the representation of death among children who had suffered losses due to COVID-19 to assess if such losses had changed their conception of death.

## Data Availability

Not applicable.
